# Development and Usability Testing of a Web-Based Workplace Disability Disclosure Decision Aid Tool for Autistic Youth and Young Adults: Qualitative Co-design Study

**DOI:** 10.2196/44354

**Published:** 2023-04-27

**Authors:** Vanessa Tomas, Shaelynn Hsu, Shauna Kingsnorth, Evdokia Anagnostou, Bonnie Kirsh, Sally Lindsay

**Affiliations:** 1 Rehabilitation Sciences Institute University of Toronto Toronto, ON Canada; 2 Bloorview Research Institute Holland Bloorview Kids Rehabilitation Hospital Toronto, ON Canada; 3 Department of Occupational Sciences & Occupational Therapy University of Toronto Toronto, ON Canada; 4 Department of Pediatrics University of Toronto Toronto, ON Canada

**Keywords:** autism, decision aids, co-design, disability disclosure, employment, knowledge translation, patient-oriented research, qualitative, usability testing, youth and young adults

## Abstract

**Background:**

Deciding whether and how to disclose one’s autism at work is complex, especially for autistic youth and young adults who are newly entering the labor market and still learning important decision-making and self-determination skills. Autistic youth and young adults may benefit from tools to support disclosure processes at work; however, to our knowledge, no evidence-based, theoretically grounded tool exists specifically for this population. There is also limited guidance on how to pursue the development of such a tool in collaboration with knowledge users.

**Objective:**

This study aimed to co-design a prototype of a disclosure decision aid tool with and for Canadian autistic youth and young adults, explore the perceived usability of the prototype (usefulness, satisfaction, and ease of use) and make necessary revisions, and outline the process used to achieve the aforementioned objectives.

**Methods:**

Taking a patient-oriented research approach, we engaged 4 autistic youths and young adults as collaborators on this project. Prototype development was guided by co-design principles and strategies, and tool content was informed by a previous needs assessment led by our team, the autistic collaborators’ lived experiences, considering intersectionality, research on knowledge translation (KT) tool development, and recommendations from the International Patient Decision Aid Standards. We co-designed a web-based PDF prototype. To assess perceived usability and experiences with the prototype, we conducted 4 participatory design and focus group Zoom (Zoom Video Communications) sessions with 19 Canadian autistic youths and young adults aged 16 to 29 (mean 22.8, SD 4.1) years. We analyzed the data using a combined conventional (inductive) and modified framework method (deductive) analysis to map the data onto usability indicators (usefulness, satisfaction, and ease of use). Grounded in participants’ feedback, considering factors of feasibility and availability of resources, and ensuring tool fidelity, we revised the prototype.

**Results:**

We developed 4 categories pertaining to the perceived usability of and participant experiences with the prototype: past disclosure experiences, prototype information and activities, prototype design and structure, and overall usability. Participant feedback was favorable and indicative of the tool’s potential impact and usability. The usability indicator requiring the most attention was ease of use, which was prioritized when revising the prototype. Our findings highlight the importance of engaging knowledge users throughout the entire prototype co-design and testing processes; incorporating co-design strategies and principles; and having content informed by relevant theories, evidence, and knowledge users’ experiences.

**Conclusions:**

We outline an innovative co-design process that other researchers, clinicians, and KT practitioners may consider when developing KT tools. We also developed a novel, evidence-based, and theoretically informed web-based disclosure decision aid tool that may help autistic youth and young adults navigate disclosure processes and improve their transitional outcomes as they enter the workforce.

## Introduction

### Background

Disclosure of a disability (ie, diagnosis, characteristics, and workplace needs) is often required to receive accommodations and supports at work. Deciding whether and how to disclose an invisible disability such as autism in a work context is complex. This process is particularly complicated for youth and young adults who are newly entering the workforce; are still developing important self-determination, advocacy, and decision-making skills [1,2]; and are at a vulnerable transitional period where outcomes could affect self-esteem and career trajectories [2,3]. Autistic youth and young adults (we use identity-first language as per recommendations by the Autism Alliance of Canada; the Autistic Self Advocacy Network, United States; and autism language commentary by Bottema-Beutel et al [4]) display varying workplace needs and disclosure knowledge and skills [5] and may benefit from tools to help navigate disclosure processes [6]; however, there is a lack of evidence-based, theoretically informed disclosure tools designed specifically for this population. This paper reports on the co-design process and usability testing of a workplace disclosure decision aid tool prototype for autistic youth and young adults in Canada.

### Autism Disclosure

Autism is a developmental disability that is characterized by difficulties with communication and social skills and exhibiting repetitive patterns of behavior and interests [7]. Employers have identified potential benefits of hiring autistic persons, such as honesty, productivity, high-quality work outputs, low absenteeism, and reliability [8,9]; however, autistic traits may also hinder opportunities and experiences, for instance, because of misalignment with the job environment, roles, and an overall lack of support at work [10]. Disclosure, which is defined as divulging diagnostic information, related traits, or workplace needs to someone at work [11], provides an opportunity to receive accommodations or adjustments that support daily workplace functioning and may enhance productivity and inclusivity [12-14]. Owing to autistic traits not being *physically* visible [14,15], autistic employees sometimes camouflaging their autistic traits in the workplace [16], or a lack of general knowledge about autistic features among workplaces [12,17], autism is often not immediately apparent, and thus, autistic persons have the choice of whether to disclose. Autistic persons must consider numerous factors when making this decision.

Among the literature on autistic adults, influencing factors include the workplace culture and environment, characteristics of the disclosure recipient, their own autistic traits and related needs, co-occurring health conditions, personal disclosure goals, past disclosure experiences, and potential outcomes of disclosure [5,12-14,18,19]. Not all outcomes are positive, such as bullying and discrimination, where sometimes nondisclosure is a safer option [20]; there are also instances when disclosure is irrelevant (eg, a person does not need an accommodation and keeps health information private) [13]. More recently, the influence of intersectional identities on autism disclosure has emerged [5,21,22]. Autistic employees who are part of marginalized or equity-deserving groups have discussed the amplified risk of disclosing their autism [22]. If an autistic person decides to disclose, they must then consider the logistical factors of the disclosure events, for example, deciding who to disclose to, such as their manager or coworkers; when, such as during the interview or after being hired; and how, such as sharing the diagnosis, specific autistic traits, workplace needs, or accommodation solutions [13,23].

Extant research on disclosure focuses on autistic adults primarily. Emerging research among the autistic youth and young adult literature displays similar factors considered when contemplating disclosure [14], with notable differences in disclosure knowledge, skills, and the extensiveness of fear of poor outcomes [2,5]. In fact, autistic adults have reported having adequate knowledge of the disclosure process [22,24]. These disclosure knowledge and skill gaps have been identified in the autistic young adult postsecondary literature [25] and among youth and young adults with other disabilities [23,26], suggesting potential distinctions in disclosure needs during this developmental period. Focusing research efforts on autistic youth and young adults is important as they face worse employment outcomes compared with other disability groups and are in the early stages of their careers [27]. Thus, disclosure outcomes may have a more profound impact on their confidence, self-esteem, career trajectory, and development of skills needed for future employment [1,2]. This trend is important as obtaining and maintaining employment early is a predictor of future employment in adulthood [28,29]. Related to autistic features, autistic employees may have different accommodation and support needs compared with other disability populations (eg, social-communication supports) [14], and they also face distinct challenges that may impede the navigation of disclosure [30-32].

Disclosure is a social process that requires recognizing the nature of the relationship with the disclosure recipient, how to structure and deliver the message, and reacting appropriately to responses [33]. Moreover, it is important to organize one’s thoughts, plan, self-regulate, advocate, set goals, and adapt to environmental cues to tailor preplanned responses when disclosing [31,33,34]. Difficulties with social-communication [30], executive functioning [32], decision-making, and self-determination [31] may impede the navigation of these processes, highlighting the importance of tailored disclosure supports for this population.

### Disclosure Interventions and Tools

A myriad of interventions exist that support specific components of employment for autistic persons, such as workplace social skills [35], interview skills [36], organizational skills [37], and other interventions that are multifaceted to support obtaining and maintaining employment [37,38]. Workplace disclosure tools are emerging to support persons with HIV [39] and mental illnesses in making disclosure decisions [40,41]. Although an autism employment toolkit includes a section on disclosure [42], to our knowledge, evidence-based, theoretically informed disclosure tools are limited for autistic persons.

### Knowledge Gaps and This Study

There are gaps regarding comprehensive disclosure supports available for autistic persons [6,21]. Having disclosure supports may increase the likelihood of making the most suitable choice [1]. It is also salient to develop disclosure tools for autistic youth and young adults because of their distinct developmental and transitional period, the influence of unique autistic characteristics, and seemingly different disclosure needs and skills compared with adults [5,6]. A purposefully built decision aid tool may address these gaps. Notably, there is also limited research that outlines a process to develop such a tool in collaboration with autistic people.

In this paper, we describe our findings and the process by which we (1) co-designed a prototype of a disclosure decision aid tool with and for autistic youth and young adults; (2) explored the perceived usefulness, satisfaction, and ease of using the prototype; and (3) understood the needed revisions to improve the perceived usability of the prototype. Our guiding research question was as follows: what is the perceived usability of a co-designed disability disclosure decision aid tool prototype from the perspective of autistic youth and young adults? Our tool was designed for use in competitive, integrated employment, which are jobs in the competitive labor market that are alongside employees with and without disabilities, where the employee has job growth opportunities and the salary is commensurate with efforts and qualifications [43]. People in the competitive labor market experience different stressors regarding disclosure compared with those who obtain employment and disclose via disability-hiring agencies [44].

## Methods

### Overview

**Figure 1:**
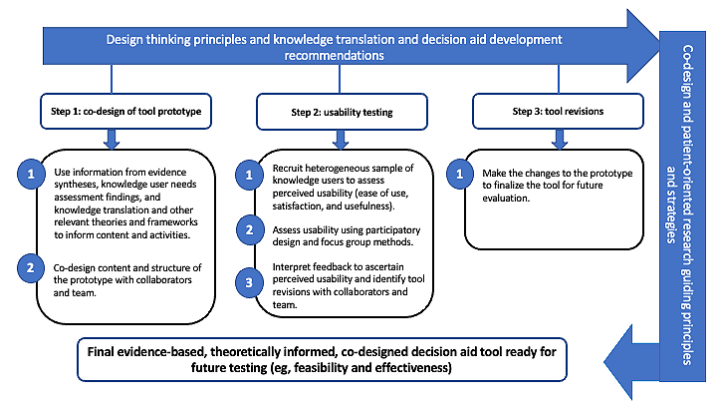
Prototype co-design and usability testing process. Before step 1, we recommend leading a needs assessment with knowledge users to understand the needs and experiences pertaining to the topic of interest. We then recommend linking experiences (if applicable) to appropriate behavior change theories and frameworks from the field of knowledge translation to identify strategies that may be incorporated into the tool.

In this section, we first outline the methods used to co-design the prototype, including the guiding principles and frameworks, team composition, and co-design steps and strategies. Next, we describe the methods used to assess the perceived usability of the prototype. The complete process used to co-design the prototype and test its usability is outlined in Figure 1.

### Conceptual Foundation and Theoretical Underpinnings

Postpositivist assumptions underpin this research study (ie, a true reality of knowledge exists but is probabilistically known and aims for objectivity) [45]. To situate this project, we turned to the field of knowledge translation (KT) following a prominent KT process model called the Knowledge-to-Action Framework [46], which guides KT activities and research. This study is within the knowledge tools stage: tailoring knowledge (primary studies, knowledge syntheses, and lived experiences) to develop an evidence-based tool. We also adopted a patient-oriented research (POR) approach [47], engaging 4 autistic youth and young adult collaborators from July 2021 to August 2022. The collaborators supported the development, evaluation of the usability of, and refinement of the prototype. They were identified via our research institution’s youth advisory council and from participants in previous studies who indicated an interest in future research partnerships.

### Step 1—Co-design of Tool Prototype

#### Overview

Co-design is an approach and philosophy that embraces the cocreation of knowledge, tools, and services with knowledge users [48]. Our aim was to co-design a decision aid tool prototype to support and enhance disclosure decision-making knowledge and skills and perceived self-determination. Decision aids are KT tools that educate knowledge users on their decisional options and outcomes and support clarification of personal values and goals to enable them to make informed decisions [49]. Workplace disclosure decision aids have been useful in reducing decisional conflict [40], increasing satisfaction with decisions [40], and enhancing the sense of control and knowledge of the decision-making process [50] among people with mental illness and mental health conditions. A decision aid tool was identified a priori by our team based on extant literature [6,12-14]. This was reinforced by the findings of our needs assessment with autistic young adults [5], which highlighted varying disclosure knowledge and skill levels, numerous factors that influence decisions, the importance of clarifying workplace needs, and the role of intersectional identities. We proposed a decision aid tool to the autistic collaborators as a solution. Notably, the co-design process was iterative and dynamic, with some preidentified considerations and steps; however, we incorporated learnings along the way.

#### Our Team

Our team comprised 4 autistic collaborators as lived experience experts and a team of researchers, trainees, and volunteers who possessed knowledge and expertise in vocational rehabilitation, workplace mental health, accommodations and disclosure, autism and neurodevelopmental disabilities, KT tool development, and the transition to adulthood. Institutional teams (ie, communications and public engagement teams) supported the graphic design and optimized the content to help people find, understand, and use the information (health literacy committee) [51]. The prototype development was led by the first author as part of her doctoral research.

#### POR Principles and Strategies

To support relationship building with the autistic collaborators and cultivate a safe, inclusive environment that embraced respect, trust, inclusivity, and flexibility, we followed the Canadian Institutes of Health Research POR Framework [52], the foundational framework [53] of engagement principles and best practices, and the engagement guidelines for youth with neurodevelopmental disabilities [54] and autistic adults [55]. To minimize power imbalances, the initial meeting began with introductory worksheets [56] to highlight interests and strengths and clarify project roles, as well as emotional check-ins or icebreakers at the beginning of subsequent meetings (eg, favorite type of coffee or tea and rating how they felt that day using a scale of animal images). Collaborators identified the prototype development tasks that they were most interested in during the first meeting; however, the types of tasks and level of involvement changed as collaborator availability and interests shifted (this was revisited at each meeting). We offered flexible Zoom (Zoom Video Communications) [57] meeting times (evenings and weekends) and formats for providing feedback. For instance, some collaborators preferred to provide feedback over email and needed structured questions to guide the feedback process. If participants had competing demands, there was flexibility in terms of their responsibilities, and the first author held separate meetings for those unable to attend group meetings. The documents and materials that we developed and used included meeting agendas, meeting slides, a plain-language project introduction document, a “what is a decision-aid tool” document, and partnership and meeting ground rules.

#### Tool Content Co-design Process

To guide the design process, we followed the 5 principles of design thinking—empathize, define, ideate, prototype, and test [58,59]—and augmented the process with POR strategies [53,54]. The design process began with a needs assessment (empathize), which is reported elsewhere [5]. The define and ideate stages comprise generating design ideas and solutions. The prototyping stage took place from August 2021 to January 2022 via Zoom and work between meetings. We used participatory strategies aligned with POR principles to co-design the prototype with the collaborators, including prompts, voting, meetings, brainstorming discussions, composite case profiling, and story sharing and capture [58,60,61]. For example, we used composite case profiling to create disclosure case scenarios based on the collaborators’ lived experiences and story sharing and capture to generate lived experience quotes. We provided structured questions that supported collaborators in generating quotes or reflecting on some of the topic areas, and they led the development of the case scenarios.

To inform prototype content and structure, we followed recommendations from the International Patient Decision Aid Standards [62,63] and KT tool development literature (eg, using theories and frameworks and knowledge syntheses and conducting needs assessments) [64]. Content was informed primarily by information and evidence from knowledge syntheses on disclosure and autism in adults [14,19], disclosure more broadly (ie, the disclosure and concealment process continuum) [23], our team’s previous needs assessment [5], and the consideration of intersectionality [65]. Self-determination theory [66] and KT theories and frameworks [67,68] also guided the content but mainly the strategies used to present information. In our previous needs assessment [5], we used a KT behavior change theory and framework to understand the roots of disclosure behaviors. We mapped the data onto domains from the Theoretical Domains Framework (TDF). The TDF domains (eg, knowledge, emotions, and beliefs about consequences) connect to intervention functions from the Behavior Change Wheel and behavior change strategies [67,68], which we used to identify important topics and strategies to present and explore content. For instance, the TDF domain “Social Influences” connects to the intervention functions of environmental restructuring, modeling, and enablement. Thus, we included case scenarios, tips, and quotes from our collaborators to display relatable real-world examples and reflection questions to explore the qualities of the disclosure recipient and the social goals of disclosure. All content and embedded strategies were reviewed, revised, and sometimes led by our collaborators. Please see Table 1 for more examples.

**Table 1:** Theoretical and conceptual foundations of the prototype—examples and strategies incorporated.

Theory, framework, or concept and construct of constructs examples		Examples of strategies and techniques to inform the prototype
**TDF^a^ and Behavior Change Wheel [67,68]**		
	TDF domain “Knowledge” links to “Education” intervention function from the Behavior Change Wheel	Information about types of disclosure, risks and benefits, disclosure goal examples, and internal and external influencing factors
	TDF domain “Behavioural Regulation” links to the “Education,” “Training,” “Modelling,” and “Enablement” intervention functions from the Behavior Change Wheel	Embedded reflection questions to self-reflect and self-monitor
	TDF domain “Emotion” links to the “Education,” “Persuasion,” “Modelling,” “Incentivization,” “Coercion,” and “Enablement” intervention functions from the Behavior Change Wheel	Experiential quotes and case scenarios
	TDF domain “Beliefs about Capabilities” links to the “Education,” “Persuasion,” “Modelling,” and “Enablement” intervention functions from the Behavior Change Wheel	Information regarding disclosure factors and logistics to build knowledge and confidence Disclosure case scenarios to model decision process Disclosure planning section and reflection questions
**Self-determination theory [66]**		
	Autonomy: feeling in control over decision-making behaviors and goals	Disclosure information and activities to support autonomous skill building (eg, reflection questions, checklists, and foundational information)
	Competence: having the necessary knowledge and skills	Foundational information, references, resources, and activities to build knowledge and skills
	Relatedness: experiencing a sense of belonging and connectedness to other people	Information about social influences and outcomes of disclosure Experiential quotes and disclosure case scenarios
**Intersectionality [65]**		
	How a person’s different identities and social categories (eg, gender, race, culture, socioeconomic status, and disability) interact in societal systems and structures of power to influence how they experience everyday life	Language and accessibility of the tool Diverse experiential quotes and case scenarios Tool section on identity and the role of intersecting identities on disclosure
**Disclosure and concealment process continuum [23]**		
	Framework that displays the various factors often considered when deciding whether to disclose for employees with nonvisible disabilities (related to oneself, others, the environment, and experiences) and logistics of disclosure and concealment (eg, strategies and timing)	Content informed using this framework (ie, factors to consider and disclosure logistics)

^a^TDF: Theoretical Domains Framework.

#### Tool Prototype

We finalized the prototype in winter 2022 (Multimedia Appendix 1): *DISCLOSURE (Do I Start the Conversation and Let on, Speak Up, and Reveal?)*. The prototype was a web-based PDF document with the following sections: (1) what is disclosure, (2) considering the workplace environment, (3) considering the person or people you are disclosing to, (4) reflecting on your needs and strengths, (5) identity and personal values, (6) disclosure in action, and (7) supplementary information and tools (1-page summary and disclosure planning worksheets). Each section contained information corroborated by research evidence and activities (ie, reflection questions and checklists), and collaborator quotes and tips were embedded throughout.

### Step 2—Usability Testing

#### Overview

This stage aligned with the testing stage in design thinking [58] and recommendations from the KT tool and decision aid development literature to assess usability [62]. Usability indicators of interest included ease of use, satisfaction, and usefulness [69,70]. Before starting this stage, with the autistic collaborators, we used a check-in document to monitor their engagement expectations, feelings, perceived contributions, and goals [71], and they identified their interests and involvement capacity.

#### Design

We used a descriptive qualitative design [72] and co-design applied via participatory design and focus group methods [60,73,74]. Descriptive qualitative design is a flexible methodology that is grounded in the principles of naturalistic inquiry and aims for higher-level understandings [72]. We used focus group questions as they are useful when designing and obtaining feedback on tools and products [75] and when collecting a range of perspectives [74]. Participatory design encompasses interactive strategies and approaches (eg, workshops) to support collaboration when designing and providing feedback on research and tools [73]. Other researchers have successfully used participatory design approaches to design and assess the usability of KT tools [76], as well as participatory design sessions and focus groups with autistic youths [77,78].

#### Participants

We used heterogeneous and snowball sampling to recruit participants [75,79]. Participants were included if they were aged 15 to 29 years [80,81]; reported having a formal autism diagnosis; were cognitively able to participate in the study session independently; were living in Canada and spoke and understood English fluently; had access to a technological device with internet; and were currently employed (full time or part time), had past competitive (paid) integrated work experience, or were currently looking for paid work. We recruited participants through a pediatric rehabilitation hospital, university-based autism social clubs, and >10 Canadian autism organizations using web-based recruitment flyers, social media posts, website postings, newsletters, listserves, and participant network connections from April 2022 to June 2022. We excluded 7 participants: we could not recontact 3 (43%), 3 (43%) were ineligible (ie, outside the age range or no autism diagnosis), and 1 (14%) dropped out. A total of 19 autistic youths and young adults participated (mean age 22.8, SD 4.1; range 16-29 years). This is an appropriate sample size given that 5 to 10 participants are deemed sufficient to assess the usability of web-based tools [82,83]. Please see Table 2 for participant demographics.

**Table 2:** Participant demographics from the prototype usability testing stage (N=19).

Demographic characteristic					Values
Age (years), mean (SD; range)					22.78 (4.1; 16-29)
**Gender, n (%)**					
	Cisgender woman			8 (42)	
	Cisgender man			8 (42)	
	Transgender man			1 (5)	
	Transgender woman			1 (5)	
	Nonbinary			1 (5)	
**Racial identity, n (%)^a^**					
	Asian—East (eg, Chinese, Japanese, and Korean)			2 (11)	
	Asian—South (eg, Indian, Pakistani, and Sri Lankan)			3 (16)	
	Asian—Southeast (eg, Malaysian, Filipino, and Vietnamese)			3 (16)	
	Black—African (eg, Ghanian, Kenyan, and Somali)			1 (5)	
	Black—Caribbean (eg, Barbadian and Jamaican)			1 (5)	
	Black—North American (eg, African Canadian)			1 (5)	
	Indigenous and First Nations			1 (5)	
	White—European (eg, English, Italian, Portuguese, and Russian)			6 (32)	
	White—North American (eg, Canadian and American)			6 (32)	
	Mixed heritage (eg, Black African and White North American)			2 (11)	
	Do not know			1 (5)	
	Other (Jewish)			1 (5)	
**Employment status, n (%)**					
	Employed full time			3 (16)	
	Employed part time			4 (21)	
	Student but with past paid work experience			5 (26)	
	Unemployed but with past paid work experience			3 (16)	
	Never been employed but interested and looking for work			4 (21)	
	**Examples of past jobs^b^**				
		Food services or hospitality	4 (21)		
		Education (eg, teacher or teaching assistant)	2 (11)		
		Software developer	2 (11)		
		Marketing	1 (5)		
		Health care (eg, nurse or research)	2 (11)		
		Stock clerk	1 (5)		
		Newspaper courier	1 (5)		
		Data entry staff	1 (5)		
		Customer service representative	1 (5)		
**Disclosed at work in the past, n (%)**					
	Yes			13 (68)	
	No			4 (21)	
	I am not sure			2 (11)	
**Other disabilities, n (%)**					
	Yes			7 (37)	
	No			9 (47)	
	I am not sure			3 (16)	
	**Examples of other disabilities^b^**				
		Social anxiety (progressive mutism)	1 (5)		
		Attention-deficit/hyperactivity disorder	4 (21)		
		Physical disability	1 (5)		
		Learning disability	1 (5)		
		Tinnitus	1 (5)		
		Obsessive-compulsive disorder	1 (5)		
		Posttraumatic stress disorder	2 (11)		
		Generalized anxiety disorder	2 (11)		
		Chronic depression	2 (11)		
		Migraines	1 (5)		

^a^Participants were asked to select all that applied.

^b^Some participants did not specify, and others noted >1.

#### Data Collection

We held 4 web-based Zoom sessions from May 2022 to June 2022. The first author (primary facilitator) and second author (secondary facilitator) led the sessions. The primary facilitator asked all the session questions, follow-ups, and prompts; led activities; took field notes; and practiced reflexivity. The secondary facilitator supported the technological aspects of the session and verbalized participants’ Zoom chat responses. We piloted session questions and technological platforms with the collaborators [74,84,85]. Before each session, participants completed a demographics survey via REDCap (Research Electronic Data Capture; Vanderbilt University) [86] and were emailed an information sheet about Zoom and Slido (Cisco Systems, Inc) [87], a password-protected link to download the prototype (via Sync; Sync.com, Inc) [88], and a document that outlined how to interact with the prototype. Participants were asked to read through the prototype once.

**Figure 2:**
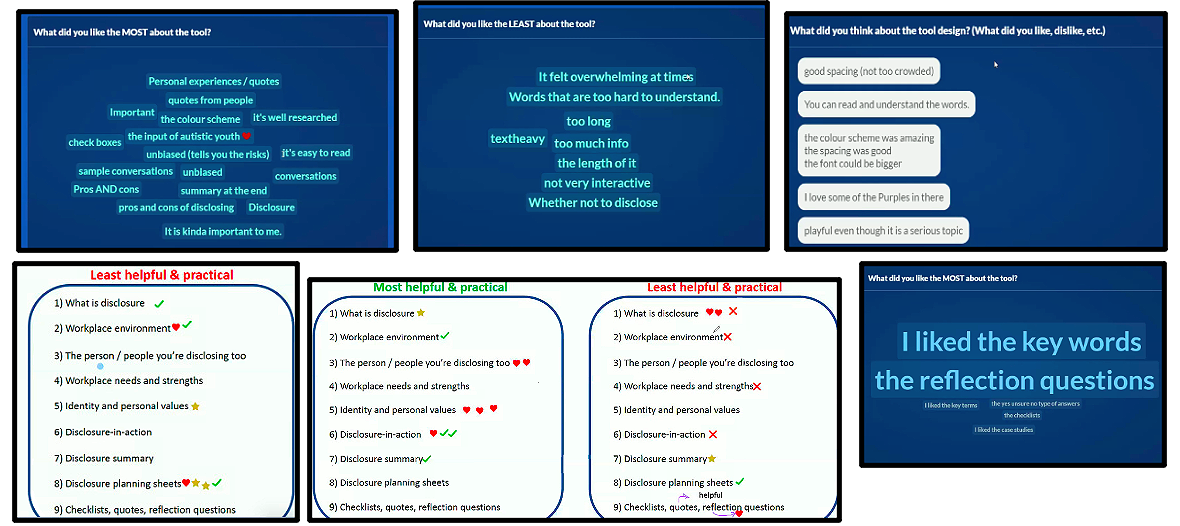
Output examples from the Slido (Cisco Systems, Inc) and Zoom Annotate (Zoom Video Communications) activities from the usability testing participatory design and focus group sessions.

We developed a flexible question guide based on recommendations by Krueger [85] and qualitative research recommendations with autistic persons (eg, opening the session with a yes or no question; Multimedia Appendix 2 [60,76,89]) [78]. We leveraged the Zoom Annotate function and used the Slido interactive platform for the session activities. The strategies we used followed participatory design approaches delineated by Stratton et al [76], Peters et al [89], and relevant literature [60]: opportunities for reflecting, visioning, voting, and ideation. Responses to the questions on Slido were anonymous and text based, and participants used the stamp or text function for the Zoom Annotate questions (Figure 2). To enhance accessibility, we emailed session questions to participants beforehand and shared the questions on Microsoft PowerPoint (Microsoft Corp) slides during the session, and participants could use the Zoom chat. We recorded the sessions, including audio, video, chat, and screen sharing, and exported data from Zoom Annotate and Slido. Session group sizes varied between 3 and 6 participants and ranged from 1 hour and 5 minutes to 1 hour and 33 minutes in duration.

#### Data Analysis

We analyzed demographic data using descriptive statistics. We used the Zoom autotranscription service to transcribe the audio from each session, and the first author verified each transcript. The first author exported and verified the text-based data. To analyze the data, we took a combined approach using conventional content analysis [90] (inductive) and modified framework method analysis [91] (deductive) in the NVivo software (version 12; QSR International) [92]. We followed the steps of familiarization, coding, developing an analytical framework, applying the analytical framework, charting data onto the framework matrix, and interpretation [91,93]. The first author led the data analysis with support from the second author. The first and second authors collaboratively coded the data from the first 2 sessions to develop the analytical framework using conventional content analysis. They applied the analytical framework to data from the third session separately, calculated intercoder reliability for each code, and discussed and resolved discrepancies when the Cohen κ coefficient was poor (≤0.4) [94,95]. The first author applied the analytical framework to data from the final session independently and charted all data onto a framework matrix (including codes, coding frequencies, definitions, speculated subcategories, and data excerpts) to create a summary of the analysis. This allowed the first author to develop superordinate categories inductively [90] while also mapping data onto a priori indicators of usability (ease of use, satisfaction, and usefulness) [69,70]. The framework matrix and interpreted categories were shared with the second, third, fourth, and senior authors, and the findings were summarized and reviewed with the autistic collaborators for feedback.

To understand the perceived usability of the tool, our team aligned our interpretations and the prevalence of participants’ positive and constructive feedback with the usability indicators of satisfaction, usefulness, and ease of use. For revisions, we considered various factors and followed guiding principles to identify and enact changes: feasibility (timeline, personnel capabilities, and technological demands), available resources (personnel and budget), and the fidelity of the tool. We implemented major suggested changes (eg, changing the structure and developing new content) if it was mentioned by participants in at least 2 sessions. We often implemented minor changes (eg, wording choices and adding minor examples) if agreed upon by the team.

#### Trustworthiness

We used various strategies to ensure study trustworthiness (credibility, transferability, dependability, and confirmability) [96,97]. To enhance credibility, all sessions were recorded, 2 coders supported the analysis, and interpretations were reviewed by team members. To ensure transferability, we compared the results with current evidence in the *Discussion* section of this paper. To improve dependability, we recruited from diverse organizations and groups and used an inductive-deductive team analytical approach. To establish confirmability, the first author practiced reflexivity, took field notes, and kept an audit trail of data collection and analytical decisions.

### Ethics Approval

We obtained institutional ethics approval to evaluate prototype usability on March 24, 2022 from the Holland Bloorview Research Ethics Board (REB 0506). The first author emailed a read-only Letter of Information and Consent Form to prospective participants and scheduled screening calls on Zoom or over the phone to relay study information, determine eligibility, and answer questions. All participants provided written electronic informed consent using REDCap [86]. We kept a restricted-access (only the first and last or senior authors), password-protected study key in our institute’s secure drive. Session transcripts were deidentified, and participant names were replaced with numbers to protect anonymity based on the study key. All data were collected using our institution’s Zoom and REDCap accounts and stored in the institution’s secure drive. Participants received a CAD $50.00 (US $36.38) electronic gift card of their choice for participating.

## Results

### Overview

We developed four categories to describe the usability testing data: (1) past disclosure experiences, (2) prototype information and activities, (3) prototype design and structure, and (4) overall usability of the prototype. The first category pertains to past experiences navigating disclosure and making disclosure decisions. The other 3 categories relate to experiences with the prototype and its perceived usability. The final category describes overall perceptions of usability. Please see Textbox 1 for participant quote examples. Participants also indicated how they interacted with the tool. Of 19 participants, 14 (74%) read through the tool once, 1 (5%) read through it more than once, 3 (16%) read the tool and also completed tool activities, and 1 (5%) had not read the tool in its entirety.

**Participant quotes from the prototype usability testing stage.**

**Category 1: past disclosure experiences**

- “I’ve pretty much made the decision on my own when it came to, like the previous jobs that I’ve held and my current jobs.” [Cisgender woman, aged 20 years, session 1]
- “I have pretty consistently disclosed my status as an autistic individual to quite a few of my past employers and including my current one.” [Cisgender man, aged 28 years, session 4]
- “I would probably go to my parents or talk to my sister if I want to. Just like talk it over with someone that would just like help me kind of see both sides...They are who I would go to for regular advice, so I think that this kind of also applies.” [Cisgender woman, aged 22 years, session 1]
- “I only tell people if it affects my day-to-day work.” [Cisgender man, aged 16 years, session 3]
- “Like for me like there’s this like sub-reddit that I like to look at. And then I like to read what other people, like, their experiences related to disclosing. Sometimes I’ll ask questions there. So, I would probably use that.” [Cisgender woman, aged 18 years, session 2]
- “I’ve partially disclosed other disabilities and parts of them, like migraines, um pain syndromes, things like that. But I’ve never disclosed my autism with an employer.” [Transgender woman, aged 28 years, session 4]

**Category 2: prototype information and activities**

- “Lack of stuff about non-disclosure, because I kind of do, thinking back on it now...there could be more about that.” [Cisgender man, aged 28 years, session 4]
- “But I really liked the checklists because, like, I like, it felt like a lot information but then the checklist was really simple.” [Cisgender woman, aged 18 years, session 2]
- “Like giving this to a friend, peer, employer, may also help them understand where you’re coming from when having a disclosure discussion.” [Cisgender woman, aged 27 years, session 4]
- “I probably wouldn’t do [the reflection questions]. It is nice to kind of like maybe just to stop and think about it, and maybe like not have the boxes because, like, I feel like that could be overwhelming and kind of daunting.” [Cisgender woman, aged 20 years, session 1]
- “Like I didn’t feel like I needed any other, any more information.” [Cisgender man, aged 25 years, session 4]
- “I kind of got the vibe that it was like kind of swaying you towards disclosing.” [Cisgender woman, aged 20 years, session 1]

**Category 3: prototype design and structure**

- “Maybe one thing that may be helpful to shorten it...might be to include appendices, where some of the like activities themselves are, and maybe these are some extra work that people can do.” [Nonbinary participant, aged 26 years, session 3]
- “It would probably be helpful to give it a division into like two different modules in and of itself. Because this would be quite chunky to go through.” [Transgender man, aged 29 years, session 3]
- “I liked the colors and the graphics that were present.” [Anonymous via Slido activity]
- “I think it could be good to have the questions on their own in the tool and then have a printable workbook with the empty spaces for people who would find filling that in helpful. It also makes it easier to go back to your answers.” [Cisgender woman, aged 20 years, session 1]
- “[Speaking of the structure in the *Disclosure-in-Action* section] The structure is fantastic in that section. I find like the way that it answers the question...It really is like well structured.” [Transgender man, aged 29 years, session 3]
- “The checklists disrupted the rhythm of reading.” [Cisgender man, aged 28 years, session 4]

**Category 4: overall usability of the prototype**

- “I haven’t seen many similar resources for older youth and young adults about disclosure...I think it’s approachable and gives a good starting point.” [Nonbinary participant, aged 26 years, session 3]
- “When I was going through it, I also found it a little bit lengthy. So, like after I was done, I felt like as I was heading towards the end, I was struggling to finish, just because I was feeling like it was a lot of information, all at once. So definitely the breaking into modules would, would really help.” [Cisgender woman, aged 24 years, session 3]
- “This accomplished its main goal, which is showing that disclosure is, like it happens on a spectrum and it’s a navigation of different factors that like come out in the balance of either helping or hurting you.” [Transgender man, aged 29 years, session 3]
- “I was kind of worried that there was going to be a lot of like really technical terms, but I thought the language was very easy to go, to read through.” [Cisgender woman, aged 22 years, session 1]
- “I found the key terms and the descriptions of like accommodations and adjustments really helpful. I think those are the section where you know, I was like, I’m learning something really new that I think I can utilize really well.” [Nonbinary participant, aged 26 years, session 3]
- “[Speaking about why *Identity and Personal Values* was the most useful] It kind of all comes back to you, what do you personally value, what do you want to get out of it and, like how you feel about your identity, because if you’re like not secure with your autism, you might not want to disclose it.” [Cisgender woman, aged 20 years, session 1]

### Category 1—Past Disclosure Experiences

This category encapsulates the past disclosure experiences of participants and the strategies and supports they have used to make disclosure decisions in employment and nonemployment settings (ie, postsecondary and social settings). Disclosure settings and experiences included at work to their boss, human resources, or coworkers; at postsecondary institutions to accessibility services and professors; and in social settings or clubs. Regarding strategies to make disclosure choices, participants spoke mainly about making decisions independently or seeking advice from friends, family, partners, or professionals (eg, therapist or job coach). Other supports included web-based discussion forums, employment programs, mentors, autistic friends, and web-based information. Notably, participants spoke about the lack of existing and accessible resources to support making disclosure decisions.

### Category 2—Prototype Information and Activities

This category represents participants’ perceptions of and experiences with the prototype content, information, and activities (eg, reflection questions, checklists, and planning sheets) and their suggestions for revisions to enhance usability. This category has two subcategories: (1) feedback and (2) suggestions.

#### Feedback

Participants provided positive and constructive feedback on the information and activities included in the prototype. Regarding the information, participants spoke positively about the detailed and well-researched content, explicit definitions, lived experience quotes and tips, and comprehensive overview of relevant factors in the disclosure decision-making process. In fact, a few participants stated that the prototype captured everything and no other information was needed. Some participants noted repetition in certain sections and the need for more balanced, realistic examples and scenarios, and many participants spoke about the lack of information on nondisclosure. Concerning the activities, some participants expressed positive sentiments toward the simplicity and practicality of the checklists and the helpfulness of the reflection questions. Other participants spoke to their preference for solely reading information rather than engaging in the embedded activities because of their learning style or because the open-ended nature of the reflection questions was daunting.

#### Suggestions

Pertaining to the information, participants advised adding lived experience quotes and examples of a person who has more workplace needs, including examples of when disclosure responses are unsatisfactory, reviewing all content to ensure that the information was realistic and not overly optimistic, and developing a new section about nondisclosure. With respect to the activities, participants proposed creating additional fill-in-the-blank activities to support key takeaways, explicitly indicating that all activities are optional for users, including more blank writing space to support activities and note taking, developing additional activities (eg, word search), and adding structure to the reflection questions. The session discourse also resulted in propositions of how the tool could be used in the future and other ideas beyond its intended purpose, for example, incorporating it into federal employment programs, sharing it with employers, and adapting and tailoring the content to make it occupation specific.

### Category 3—Prototype Design and Structure

This category includes participants’ perceptions of and experiences with the prototype design and structure and their recommendations to improve perceived usability. This category has two subcategories: (1) feedback and (2) suggestions.

#### Feedback

Participants mainly expressed positive remarks regarding the design and structure of the prototype, for instance, compliments about the color scheme, color contrast, graphics, spacing of text and graphics (ie, not overcrowded), and the organization and structure of the section content. Participants also provided some constructive feedback: the lack of clear places to take breaks, that the amount of text was overwhelming, and that the activities disrupted the flow when reading the tool. Although most participants spoke favorably of the color scheme, a few participants stated that the colors may not be suitable for persons who are color-blind.

#### Suggestions

Specific to the design, most participants recommended representing some of the information in different formats such as visuals, graphics, videos, and charts. For instance, participants encouraged replacing the text-heavy summary page with a visual design element of a flowchart. Regarding tool structure, most of the suggestions centered on dividing the tool into distinct parts or modules to encourage breaks and create an additional workbook or appendix that included all the activities and space to complete them. Another minor suggestion raised by a few participants focused on the consistent alignment of content on each page (eg, margins and placement).

### Category 4—Overall Usability of the Prototype

This category captures participants’ perceptions of the usability of the prototype. There are three subcategories that link directly to a priori indicators of usability: (1) satisfaction, (2) usefulness, and (3) ease of use.

#### Satisfaction

This subcategory includes participants’ perceptions of whether they liked the prototype and found it appealing and engaging. All participants shared sentiments of overall satisfaction with the tool, with only a few noting tool components that were less appealing. Participants shared their appreciation for the detailed and well-researched information, the accessibility of the language, the activities and quotes or case scenarios, the prototype’s novelty, and how the content effectively captured the disclosure process as a spectrum and individualized decision. A few participants stated that the prototype’s positive and optimistic tone made it less appealing. Although the components of the prototype that participants liked the most and least varied at the individual level, there were commonalities across sessions. Participants in most sessions indicated liking the practical and lived experience components (eg, checklists, case scenarios, quotes, and reflection questions) the most. Participants said that the tool was text-heavy and the lack of information on nondisclosure made it less appealing. Some participants noted that there was nothing they liked the least in the prototype.

#### Usefulness

This subcategory explores participants’ perceptions of whether the prototype and its components were helpful and relevant. Overall, most participants expressed that the prototype may be useful to support autistic youth and young adults in making disclosure decisions. Participants shared that the prototype introduced new and helpful information and may improve the autistic person’s disclosure decision-making confidence and offer support when making disclosure decisions in the real world, as well as that the content was comprehensible and explicit. A few participants spoke about how the prototype was less useful to them as they were already quite knowledgeable about the disclosure process. The components of the prototype that participants found to be the most and least helpful varied at the individual level; however, there were consistencies across sessions. The *Identity and Personal Values* section was continuously identified as one of the most helpful sections in each session. After *Identity and Personal Values*, the *Workplace Needs and Strengths* section was commonly identified except in 1 session. Other helpful sections and components included the *Disclosure-in-Action*, *Workplace Environment*, and *Person/People You’re Disclosing to* sections and the activities. The disclosure planning sheets and summary page in the *Supplemental Resources* section were noted as the least helpful by a few participants in each session because of the repetitiveness of the presented information. Other participants also identified the *Workplace Environment* and *What is Disclosure?* sections as the least helpful.

#### Ease of Use

This subcategory highlights participants’ experiences using and navigating the prototype and their perceptions of its accessibility and simplicity and the required effort to use it (eg, time and cognitive effort). Most participants expressed that the prototype was accessible and well organized and that the language was highly comprehensible. Very few participants felt that the activities and information were difficult to understand. There was a comment to ensure the tool’s compatibility with a screen reader. Several participants shared concerns with the length of the tool, the daunting amount of information presented at once, the lack of structure of the reflection questions, and the fact that the time and effort required to use the tool without purposeful breaks may be onerous. Some participants did note that the length was suitable and that the prototype had an adequate amount of information. Interestingly, although participants shared ideas to restructure and present information, they did not suggest removing any content.

### Tool Revisions

Primary tool revisions aimed to enhance the ease of using the prototype. For example, we divided the tool content into 3 parts; moved the reflection questions and checklists to an optional part of the tool (part 3); added structure to the reflection questions; created introductory videos for each tool part; represented content via different formats, such as graphics and a flowchart; and condensed information where appropriate. In addition, the first author and autistic collaborators crafted new content, such as a new section about nondisclosure that includes information (eg, reasons for choosing nondisclosure and check-in questions if one decides not to disclose) and illustrative nondisclosure case scenarios grounded in the collaborators’ lived experiences. We also made minor revisions. We added more collaborator quotes or tips, key takeaway questions, and new content examples (eg, adding a union representative as someone one can disclose to); ensured that the content was consistently aligned on each page; revisited word choices (ie, using *reliable* instead of *trustworthy*); and checked the PDFs to ensure screen reader compatibility. Some suggestions were not implemented owing to feasibility, ensuring fidelity to the tool goals, and low prevalence of the recommendation. For example, a participant suggested creating new sections about social skills and small talk, which were beyond the scope and goals of the tool. We removed the disclosure planning sheets and repurposed the summary page because of repetition with the reflection questions and restructuring it into a graphic flowchart, respectively. Examples of the revised tool are provided in Multimedia Appendix 1.

## Discussion

### Principal Findings and Comparison With Prior Work

Participant feedback on the tool prototype is propitious of its potential impact, usability, and the process used to develop it. Our findings highlight the importance of engaging knowledge users throughout the entire prototype co-design and testing process; incorporating co-design strategies and principles; and having content informed by relevant theories, evidence, and the needs of knowledge users. Researchers, clinicians, and KT professionals looking to co-design KT tools may consider applying or adapting the process used by our team. To our knowledge, this is one of the first evidence-based, theory-informed workplace disclosure decision aid tools specifically for autistic youths and young adults.

Our prototype design and testing process was grounded in patient engagement and co-design methods. The collaborators supported the co-design of the prototype; advised on research methods for usability testing and data interpretations; and decided on, supported, and co-led prototype revisions. In the KT tool development literature, engaging knowledge users is identified as imperative, which is also emerging in the autism literature [98]. Oftentimes, knowledge users are engaged as participants offering feedback on drafts (ie, our usability testing stage). Specific to decision aids, there are instances of knowledge users engaged as partners; however, a recent systematic review identified this as less common [99]. An update to the International Patient Decision Aid Standards highlights the importance of knowledge user engagement in decision aid development [100]. Our work is particularly valuable because it involves autistic youth and young adults throughout the development of the decision aid prototype and the research phases. The criticality of engaging knowledge users in research is evident in the literature, such as making research more relevant and meaningful, empowering knowledge users engaged in the project, and enhancing the impact of research findings and outputs [101]. Autism researchers and self-advocates have identified the need to prioritize the collaboration of autistic persons in research to ensure that research goals and outputs are impactful and meaningful [102].

We followed guidance from the KT tool development literature, decision aid development recommendations, and design thinking principles to develop the prototype. This included using theories and frameworks and knowledge syntheses, conducting a needs assessment, prototyping, and testing the usability of the prototype. It is essential to follow evidence-based recommendations and principles when co-designing KT tools to ensure that they are grounded in evidence and knowledge users’ needs and that the design process encompasses necessary and foundational design and engagement principles [48,62]. Although the importance of theory is discussed in the KT tool development literature, minimal examples exist to showcase the pragmatics of how this is done. Furthermore, the literature highlights how theory incorporation is lacking in the process of developing decision aid tools specifically [103,104] and that decision aid developers would be wise to consider and incorporate theories throughout the development process, particularly KT theories that support behavior change (eg, TDF) [103].

Although revisions are required to enhance the usability of the prototype (ie, mostly ease of use), overall feedback was positive and promising. Constructive feedback aligned with the literature regarding the needs of autistic people, for example, having difficulties with open-ended questions [55], the importance of diverse representations of information, and incorporating learning breaks to support information processing [105]. Challenges with ease of use in particular were also displayed in the prototype testing of a health care toolkit for autistic adults [98]. Our results highlight the importance of developing KT tools that consider (both in content and structure) the distinct and diverse needs of autistic persons. Positive feedback aligns with the literature and recommendations for electronic tools for autistic users [55], such as using plain language, providing concrete examples and cases, using graphics, and defining key terms. Notably, although there were consistencies across the participants’ feedback, there were also individual variations, specifically, when participants identified the tool components that they liked the most and least and found the most and least helpful and the features that made the tool easy to use. This speaks to the individualized nature of the disclosure process, varying knowledge levels of the disclosure process, different learning styles (ie, those who preferred activities vs those who preferred reading information), and the heterogeneity of autistic youth and young adults. Tools developed for autistic persons should consider inclusive design principles that acknowledge unique learning differences, account for individualized preferences and needs, and ensure that takeaways can be personalized to meet the needs and goals of the individual [98]. Although the revised tool requires further testing to understand its impact, prototype feedback shows immense promise to be a satisfactory and useful tool.

### Limitations

It is important that participants who are assessing product usability are familiar with the product. Despite stipulating that participants must review the prototype before the session, it is difficult to guarantee this, and participants varied in how they interacted with it. However, this variation is reflective of real-world experiences where some people review content and activities in depth and others prefer a cursory glance. All experiences and perspectives are important to capture. Second, self-selection bias may be a limitation given that those who had disclosure experience or were more passionate about the topic may have been more likely to participate. Finally, this tool was developed from Western ways of knowing and doing (ie, incorporated evidence and participant sample; only 1/19, 5% of the participants identified as Indigenous or First Nations) and does not consider or incorporate Indigenous worldviews (eg, perceptions of disability, strengths, or gifts, and how disclosure as an act is perceived). There is strength in understanding and reflecting on diverse ways of knowing and doing, particularly in the Canadian context; however, this would require a different research approach given the divergence between assumptions and beliefs in Indigenous and Western research paradigms and ontologies [106].

### Future Research and Implications

First, aligning with the decision aid development literature [62], we suggest that future studies evaluate the usability of the revised tool, assess other important outcomes such as tool feasibility, and collect data on perceived impact. These studies should aim to recruit more diverse perspectives, for instance, specific to gender as this study predominantly included cisgender women and cisgender men. This is the next step identified by our team. Furthermore, more in-depth user experiences could be obtained qualitatively once the tool is finalized to better understand overall user experiences and how the tool may be used in practice. Second, future studies could assess the tool from the perspective of professionals who support clients with disclosure decision-making, such as vocational rehabilitation professionals, career counselors, occupational therapists, and psychologists. This feedback may help us understand how the tool can be used to complement their existing practices, processes, and programs and identify important missing information. Furthermore, feedback from employers may provide insights into organization-specific disclosure processes and how this tool could be an embedded resource or to provide additional information to be included in the tool from an employer perspective. Finally, although this tool is intended for autistic youth and young adults, future studies could explore whether it is useful for autistic adults and older adults, in particular those with a recent autism diagnosis as age of diagnosis may influence disclosure [14].

### Conclusions

We developed a comprehensive, evidence-based, and theory-informed workplace disclosure decision aid tool for autistic youth and young adults. Our study illustrates the critical importance of engaging knowledge users to co-design and test the prototype; ground tool content in evidence and knowledge users’ needs; and use relevant theories, models, and frameworks. Given the positive findings and feedback obtained that indicate perceived, adequate usability upon prototype revisions, our co-design process may be useful for KT practitioners, clinicians, and researchers who are developing KT tools. After further testing and refining, this tool may be used by autistic youth and young adults in Canada to support disclosure decision-making processes at work.

## Appendix

Multimedia Appendix 1 Examples from the tool prototype and examples from the revised tool.

Multimedia Appendix 2 Usability focus group and participatory design session questions.

## Abbreviations

KT: knowledge translation
POR: patient-oriented research
REDCap: Research Electronic Data Capture
TDF: Theoretical Domains Framework

## Data Availability

The data sets generated and analyzed during this study are not publicly available because of institutional ethics guidelines and the constituents of the approved ethics application. The data generated may be made available from the last and senior author (SL) conditional upon approval from our institution’s ethics board.
