# Supplementary material for: Development and Usability Testing of a Web-Based Workplace Disability Disclosure Decision Aid Tool for Autistic Youth and Young Adults: Qualitative Co-design Study
Source: JMIR Form Res. 2023 Apr 27;7:e44354. doi: 10.2196/44354 (PMC10176134; doi:10.2196/44354)
Supplement: Multimedia Appendix 1 [file formative_v7i1e44354_app1.docx]

## Multimedia Appendix 1

### Examples from the Tool Prototype

***
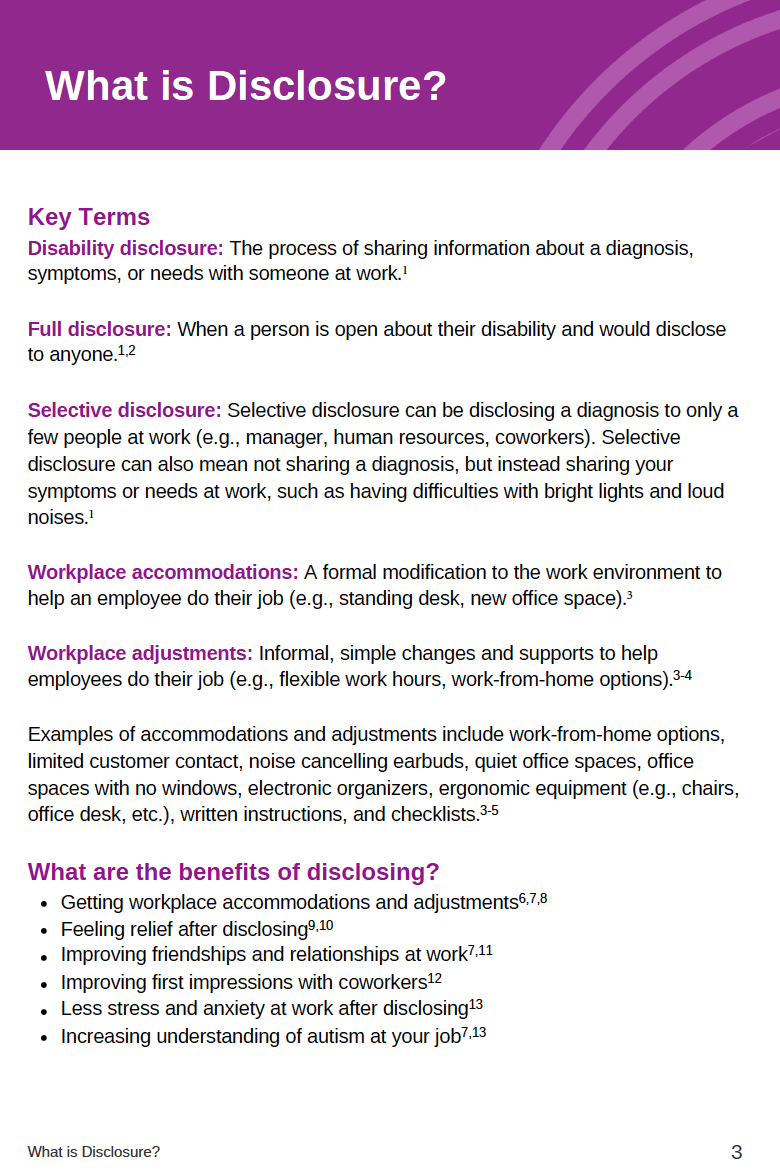
***

***
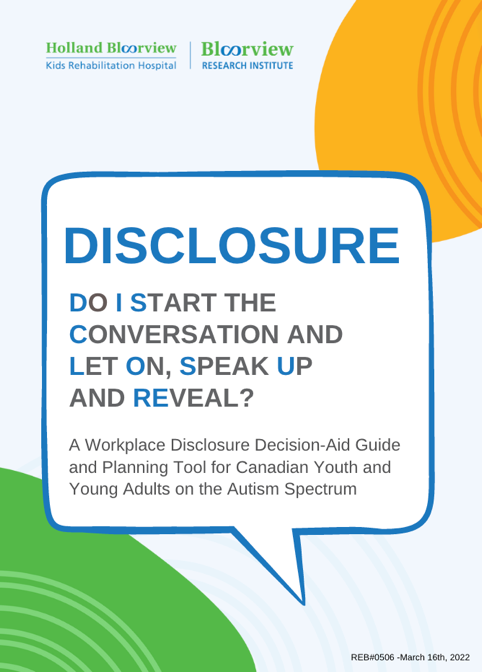
***

***
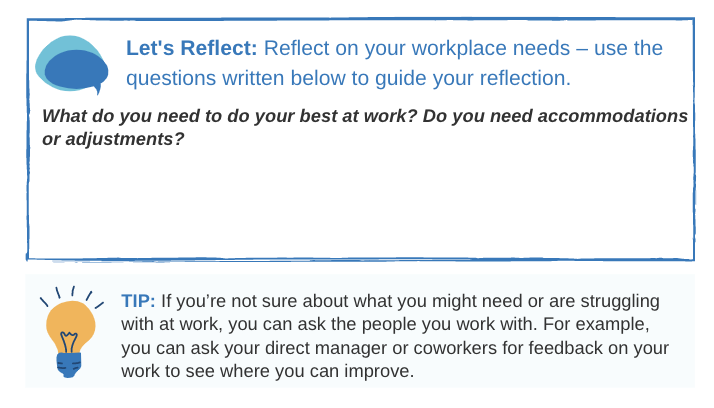
***

**
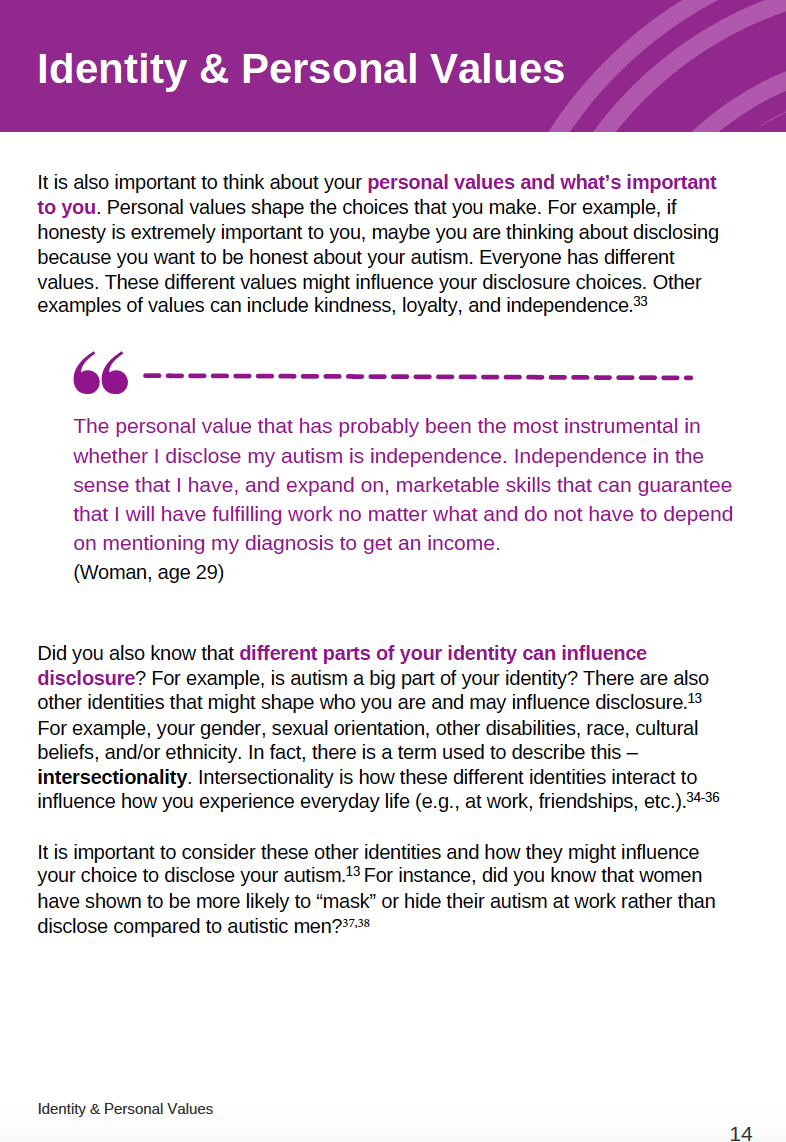
**

***
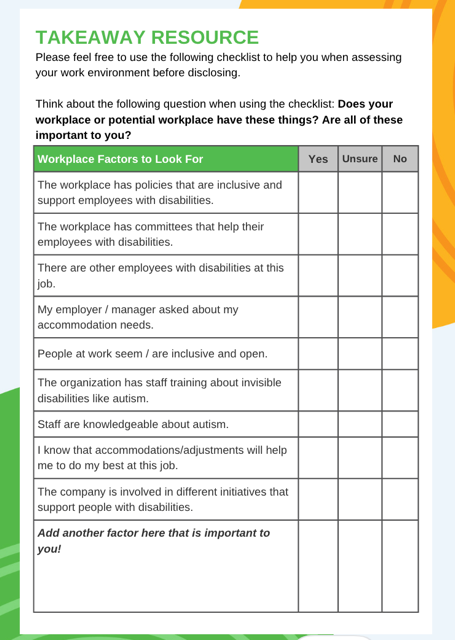
***

### Examples of Revised Tool

#### New non-disclosure section, enhanced graphics, structured reflection questions, separating tool into three parts, etc.


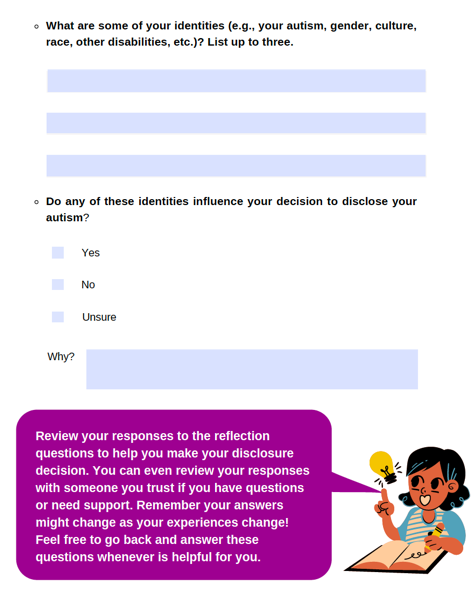


***
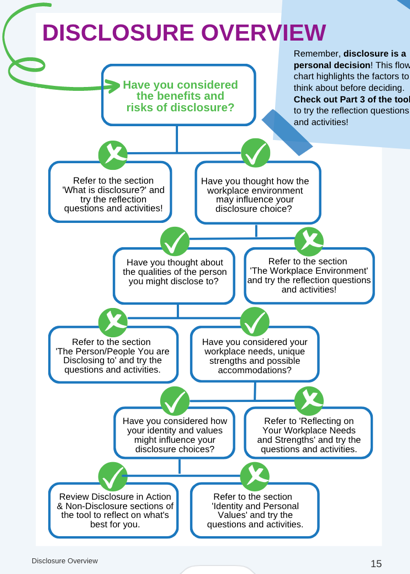

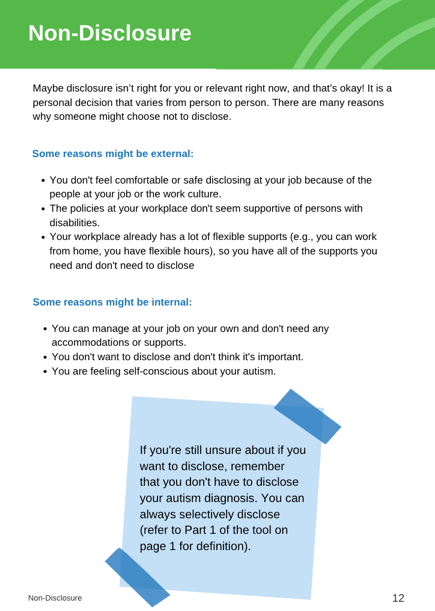
***

***
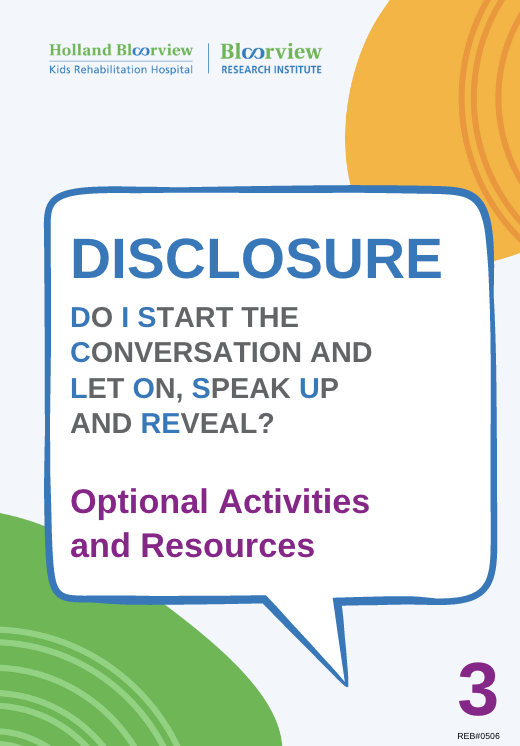

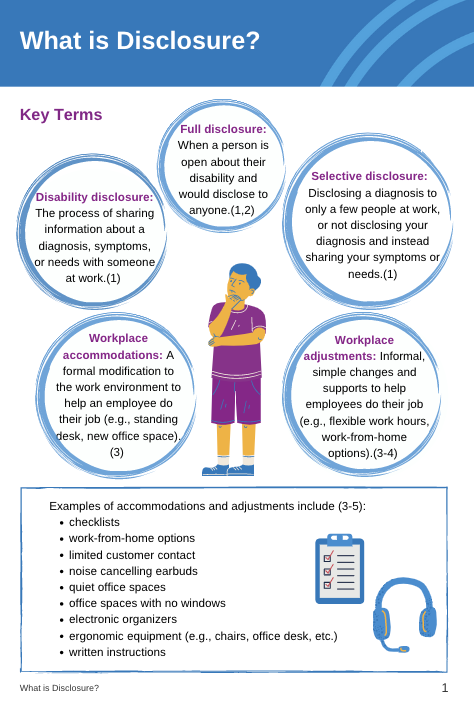
***
