# Supplementary material for: Development and Usability Testing of a Web-Based Workplace Disability Disclosure Decision Aid Tool for Autistic Youth and Young Adults: Qualitative Co-design Study
Source: JMIR Form Res. 2023 Apr 27;7:e44354. doi: 10.2196/44354 (PMC10176134; doi:10.2196/44354)
Supplement: Multimedia Appendix 2 [file formative_v7i1e44354_app2.docx]

## Multimedia Appendix 2

### Usability Focus Group and Participatory Design Session

#### Opening question

1. This first question, I’d like you to answer yes or no. Have you disclosed to someone at work before? This could include your employer/boss, manager, and/or coworkers. (*This question answered using Zoom – either unmuting microphone or chat function*)

#### Introductory/transition question

1. Can you describe any supports that you’ve used in the past when deciding to disclose at work? (*Aligns with sensitizing approach used in participatory design – i.e., reflecting on one’s past experiences)* (*This question answered using Zoom – either unmuting microphone or chat function*)
   1. PROBE: People, online resources, toolkits, your own research, etc.

#### Key questions

You were asked to review a draft of a tool that we developed to help youth and young adults on the autism spectrum make disclosure decisions at work. As a reminder the tool includes the following sections after the table of contents and tool introduction: 1) what is disclosure, 2) considering the workplace environment, 3) considering the person/people you are disclosing to, 4) reflecting on your needs and strengths, 5) identity and personal values, 6) disclosure and non-disclosure in action, 7) summary, and 8) disclosure planning.

**Primary facilitator shares the tool prototype in the Zoom chat and shares screen to re-orient participants to the tool*

1. First, we’re going to talk about some of your experiences reviewing and using the tool. (Usability indicator - Ease of use) (*This question answered using Zoom – either unmuting microphone or chat function*)
   1. Let’s start with how you interacted with it. For example, did you read it once, answer the reflection questions, etc.? Please describe.
   2. Consider the amount of time you spent using the tool – was the time appropriate given the tool’s purpose? Did you find the tool too long, too short, or just right?
   3. Was the tool easy to read and work through? For example, how did you find the flow of the tool including the content and interactive sections?
   4. Did the tool provide you with enough information and was everything explained well (e.g., definitions of terms, concepts, etc.)?
2. Do you think the tool is helpful to support autistic youth and young adults make disclosure decisions at work? (Usability indicator - Usefulness) (*This question answered using Zoom – either unmuting microphone or chat function*)
   1. Please describe why or why not
   2. Does the tool offer diverse options and perspectives so that you can fairly decide and make your own disclosure decisions? (E.g., does not sway you in one direction)
3. Reflect on your overall thoughts about the tool (Satisfaction). Think about what might make the tool more engaging and appealing (heart) (from Peters et al., 2018 connecting to heart, hand, and mind method).
   1. What did you like most about the tool? *Question answered on Slido to generate a Word Cloud.*
   2. What did you like least about the tool? *Question answered on Slido to generate a Word Cloud.*
   3. PROBE: Let’s chat about some of your responses. Does anything need to be added, removed, and/or changed to make the tool more engaging and appealing?
4. What did you think about the design of the tool (e.g., colours, layout, examples of shapes and graphics used)? (*Question answered on Slido using open-text option).*
   1. What did you like? Dislike?
   2. Do you have other design ideas for the tool?
   3. PROBE: Let’s chat about some of your responses.
5. Reflect on the different sections of the tool. Think about what might make the tool more helpful (mind) and practical (hand) (from Peters et al., 2018 connecting to heart, hand, and mind method). (*Answered using Zoom annotate or unmuting microphone or chat function depending on comfort*)
   1. Which components of the tool were most helpful and practical? Which components of the tool were least helpful and practical? Write / stamp your response using the Zoom annotate function on the slide.
   2. Let’s chat about some of your responses. Do you think anything needs to be added, removed, and/or changed in the tool to make it more helpful and practical?

#### Ending question

1. Think about the ideal tool or support that would help you make disclosure decisions (*visioning technique from participatory design*). Based on this, is there anything else you want to add that you think is important for us to know as we continue to develop the tool? (*This question answered using Zoom – either unmuting microphone or chat function).*
   1. Was there anything that you were expecting to be in the tool that wasn’t there?
